# A Case of Muscle-Invasive Bladder Cancer With Pelvic Lymph Node Involvement Treated With Pembrolizumab and Subsequent Radical Cystectomy and Maintained No Evidence of Disease After Surgery

**DOI:** 10.7759/cureus.19375

**Published:** 2021-11-08

**Authors:** Shinichi Takeuchi, Keita Nakane, Chiemi Saigo, Tatsuhiko Miyazaki, Takuya Koie

**Affiliations:** 1 Urology, Gifu University, Gifu, JPN; 2 Pathology, Gifu University, Gifu, JPN; 3 Department of Diagnostic Pathology, Gifu University Hospital, Gifu, JPN

**Keywords:** surgery, pembrolizumab, chemotherapy, lymph node involvement, muscle-invasive bladder cancer

## Abstract

A 67-year-old man was referred to our hospital with chief complaints of macrohematuria and anemia. He was diagnosed with muscle-invasive bladder cancer (MIBC) with right external iliac lymph node (LN) involvement and received two courses of gemcitabine and carboplatin. After chemotherapy, left external iliac LN involvement was identified as a new lesion, even though the bladder cancer (BCa) and right external iliac LN decreased in size. Therefore, pembrolizumab was administered as a second-line treatment. The bladder tumor and positive LNs subsequently shrunk. Open radical cystectomy and bilateral ureterocutaneostomy were also performed. The pathological examination of the surgical specimen indicated urothelial carcinoma, pathological stage Tis, and negative LN involvement. The patient was followed up for 30 months without evidence of local recurrence or distant metastasis.

## Introduction

Radical cystectomy (RC) is the definitive treatment option for patients with muscle-invasive bladder cancer (MIBC) [1]. However, the optimal treatment for MIBC patients with clinically evident lymph node (LN) involvement remains debated [2]. Although pembrolizumab as a second-line treatment significantly improves the overall survival (OS) in patients with platinum-refractory advanced urothelial carcinoma (UC) [3], the US Food and Drug Administration recently approved pembrolizumab monotherapy as a first-line treatment in platinum-ineligible patients, regardless of tumor PD-L1 expression [4,5]. Currently, the PURE-01 trials have shown its efficacy and safety in patients with negative LN MIBC who underwent neoadjuvant pembrolizumab followed by RC and pelvic lymph node dissection (PLND) [6,7].

Herein, we report a patient with MIBC and positive LN who underwent RC with PLND after pembrolizumab administration as a second-line therapy that led to the shrinkage of the bladder cancer (BCa) and positive LNs and consequently had no evidence of disease two years after surgery.

## Case presentation

**Figure 1:**
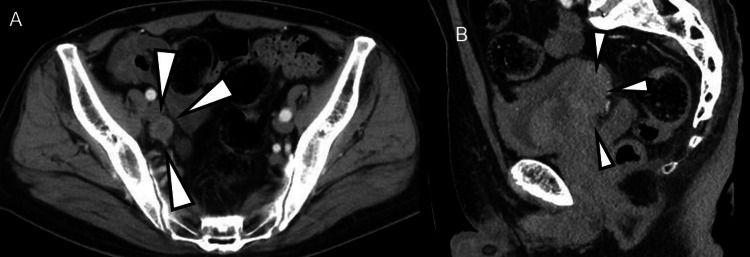
Bladder cancer and lymph node involvement at the time of the initial diagnosis (A) Computed tomography revealed left hydronephrosis, a bladder tumor on the right lateral wall, and right external iliac LN involvement at the time of the initial diagnosis. (B) T2-weighted magnetic resonance imaging prior to our therapy revealed a bladder tumor with invasion of the surrounding fibroadipose tissue at the right lateral wall before chemotherapy.

A 67-year-old man with chief complaints of macrohematuria and an abnormally low hemoglobin level (4.8 g/dL; normal range, 13.7-16.8 g/dL) was referred to our hospital. His performance status was zero and none of any co-morbidities were identified. Whole-body computed tomography (CT) revealed left hydronephrosis, a bladder tumor on the right lateral wall, and right external iliac LN involvement (Figure 1A). CT revealed a bladder tumor with invasion of surrounding fibroadipose tissue on the right lateral wall (Figure 1B). Transurethral resection of the bladder tumor was performed after blood transfusion; histopathological diagnosis revealed high-grade UC of the bladder with muscle layer invasion. BCa was classified as clinical T3bN1M0 according to the staging system defined in the American Joint Committee on Cancer Staging Manual [8]. His estimated glomerular filtration rate (eGFR) was 64.57 mL/min and his renal function was maintained at a normal eGFR level during the medication for BCa. He received two combined courses of gemcitabine and cisplatin (GC; 1,000 mg/m^2^ gemcitabine on days 1, 8, and 15, and 70 mg/m^2^ cisplatin on day 2) every 21 days. To monitor the treatment effect on BCa, the patient underwent whole-body CT and pelvic MRI after every two courses of systemic therapy.

**Figure 2:**
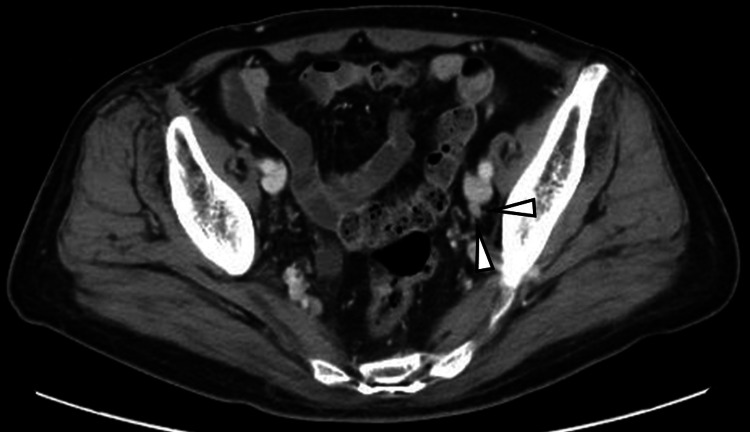
Left external iliac lymph node involvement as a new lesion After two courses with chemotherapy, computed tomography revealed left external iliac lymph node involvement as a new lesion, although the bladder cancer and right external iliac lymph node decreased in size.

**Figure 3:**
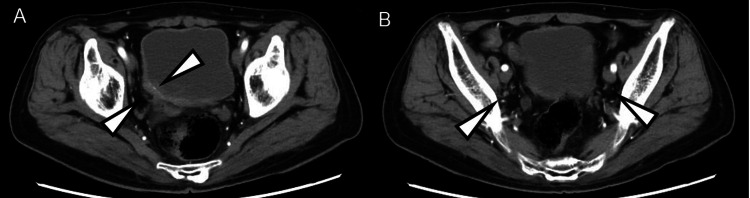
After the administration of pembrolizumab After the administration of three courses of pembrolizumab, a bladder tumor (A) and positive lymph nodes (B) shrunk before starting the therapy.

**Figure 4:**
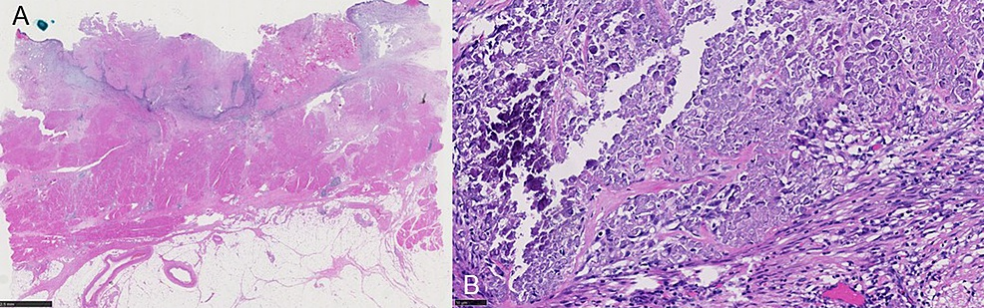
Histopathological findings Pathological examination of the surgical specimen indicated urothelial carcinoma, pathological stage Tis (A) and negative lymph node involvement (B).

After two courses with GC, CT revealed left external iliac LN involvement as a new lesion, although the BCa and right external iliac LN decreased in size. Disease progression was diagnosed according to the Response Evaluation Criteria in Solid Tumors guidelines, version 1.1 [9] (Figure 2). It was difficult to explain why the left external LN has enlarged even though other lesions showed a positive effect after GCarbo. One possibility could be that the UC being a heterogeneous tumor, may have unique properties in this case. As a second-line treatment, pembrolizumab (200 mg) was administered every three weeks. Grade 4 macrohematuria according to the Clavien-Dindo classification [10] occurred two weeks after the administration of pembrolizumab; thus, he immediately underwent endoscopic hemostasis for the bleeding site after blood transfusion. The break-through bleeding occurred from the surface of the BCa. Thereafter, the patient received two more courses of pembrolizumab. CT findings after the administration of pembrolizumab revealed shrinking of the BCa, positive LNs, and right kidney (Figure 3A-3B). Three weeks after the final dose of pembrolizumab, open RC with bilateral pelvic lymphadenectomy and bilateral ureterocutaneostomy were performed because of the possibility of palliative surgical treatment. There was firm adhesion between the right lateral wall of the urinary bladder and the pelvic floor. The adhesion around the bilateral pelvic lymph node was not identified. There have been no surgery-related complications. The pathological examination of the surgical specimen by two pathologists at our institution indicated UC, with a pathological stage of Tis without lymphovascular invasion, a negative surgical margin, and negative LN involvement (Figure 4A-4B). The patient was followed up for 30 months without evidence of local recurrence or distant metastasis.

## Discussion

According to the guidelines of the National Comprehensive Cancer Network, chemoradiotherapy has better oncological outcomes for MIBC with positive LN involvement than chemotherapy being used alone [10]. Several studies have recently demonstrated that the combination of chemotherapy and surgery showed better local disease control and oncological outcomes in MIBC patients with regional positive LN compared with single treatment modalities such as palliative chemotherapy and surgery alone [2,11]. In the KCSG GU 17-03 trial, the MIBC patients with N1 who underwent both RC and chemotherapy had a significantly improved OS compared with those who received RC or chemotherapy alone (P < 0.001) [2]. Galsky et al. reported the oncological outcomes of 1,739 MIBC patients with clinical LN involvement [12]. According to their study, patients who received combined modality therapy, such as RC and pre-/post-chemotherapy, achieved durable disease control and long-term survival compared with those who underwent RC or chemotherapy alone [11]. Therefore, induction chemotherapy for MIBC patients with clinical LN involvement may have potential benefits for OS [12].

According to the phase III KEYNOTE-045 trial, second-line pembrolizumab significantly improved OS (P = 0.002) and resulted in a lower rate of treatment-related adverse events than chemotherapy for advanced UC [3]. In phase II KEYNOTE-052 trial, first-line pembrolizumab had antitumor activity and acceptable tolerability in patients with cisplatin-ineligible UC [5]. In addition, neoadjuvant pembrolizumab followed by RC with PLND may have potential benefits, including surgical safety and maintenance of the immunotherapy effect after surgery, in patients with non-metastatic MIBC [6,7]. Conversely, the addition of pembrolizumab to first-line platinum-based chemotherapy did not significantly improve the oncological outcomes in patients with metastatic UC, based on the KEYNOTE-361 trial [13]. Therefore, platinum-based chemotherapy is currently the standard treatment for patients with metastatic MIBC. However, RC with PLND may be a treatment option to improve survival in MIBC patients with positive LN involvement, if shrinkage of the primary BCa or LN is obtained after the administration of cisplatin-based chemotherapy followed by pembrolizumab. Further large-scale clinical or case observational studies are warranted.

## Conclusions

The present case report shows that pembrolizumab as a second-line may have a potential benefit in patients with MIBC and LN involvement who underwent RC. Therefore, we suggest that RC is one of the treatment options in patients with MIBC and LN involvement who have achieved shrinking of the primary lesion and/or LN metastases after the administration of pembrolizumab.
